# Supplementary material for: Stool antigen-based enzyme immunoassays: performance evaluations and automation
Source: J Clin Microbiol. 2026 Jun 12;64(7):e00006-26. doi: 10.1128/jcm.00006-26 (PMC13343952; doi:10.1128/jcm.00006-26)
Supplement: Supplemental tables and figure — Tables S1 to S5 and Fig. SF1. [file jcm.00006-26-s0001.docx]

| **Supplemental TABLE S1** EIA OD cutoff value for *Cryptosporidium* positive compared to microscopic method (n=41) | | | | | |
| --- | --- | --- | --- | --- | --- |
| Method | Detected | Non-detected | Sensitivity (%) | Specificity (%) | EIA OD cutoff for positive detectability (450/620nm) * |
| DFA/EIA | 21 | 20 | 100 | 100 | >=0.09 |
| Microscopic (wet mount) | 15 | 26 | 71.4 | 100 | >=0.259 |

* DFA was the reference method; OD: Optical Density (450/620 nm).

**Supplemental TABLE S2** *Giardia* EIA compared to *Giardia* microscopy

|  | |  | Microscopy (n=84) | | |
| --- | --- | --- | --- | --- | --- |
|  | |  | Positive | | Negative |
| EIA | | Positive | 34 | | 21 |
|  | | Negative | 0 | | 29 |
|  | Total | | 34 | | 50 |
|  | | (%) | | 100 (sensitivity) | 58 (specificity) |

| **Supplemental TABLE S3** Discrepant *Campylobacter* EIA samples compared to culture | | | | | | |
| --- | --- | --- | --- | --- | --- | --- |
| sample ID | Organism from culture | Campy Chek EIA (TechLab) | | Premier EIA (Meridian) | | *Campylobacter* PCR* |
|  |  | OD read | positive (+) / negative (-) | OD read | positive (+) / negative (-) |  |
| 2927D | *C. coli* | 0.003 | - | 0.010 | - | not detected |
| 8780 | *C. coli* | 0.003 | - | 0.010 | - | not detected |
| 1611 | *C. upsaliensis* | 0.003 | - | 0.070 | - | not detected |
| 27641 | (unknown**) | 0.008 | - | 0.160 | + | NA |
| 7744 | *C. jejuni* | 0.167 | + | 0.030 | - | NA |

*Verigene Enteric Pathogens Nucleic Acid Test (DiaSorin, Stillwater, MN). **The organism tracking was lost.

**Supplemental TABLE S4** Automation EIA performance compared to manual method

| EIA Platform | Target | Stool Specimen Type* | Total specimen No tested | Positive Number | | Negative Number | | Overall Agreement (%) |
| --- | --- | --- | --- | --- | --- | --- | --- | --- |
|  |  |  |  | Manual Method (Reference) | DS2 Method | Manual Method (Reference) | DS2 Method |  |
| Campy EIA | *Campylobacter* | CB-Preserved | 52 | 22 | 23 | 30 | 29 | 98.1 |
| Cryptosporidium II | *Cryptosporidium* | Preserved | 60 | 26 | 26 | 34 | 34 | 100 |
| Giardia EIA | *Giardia* | Preserved | 93 | 45 | 52 | 48 | 41 | 92.5 |
| Shiga Toxin EIA | STEC | Raw or preserved | 36 | 14 | 14 | 22 | 22 | 100 |
| H. pylori EIA | *H. pylori* | Raw | 91 | 45 | 47 | 46 | 44 | 97.8 |
|  |  | CB-preserved | 59 | 30 | 31 | 29 | 28 | 98.3 |
| LUA EIA | *Legionella* | (Urine) | 50 | 21 | 20 | 29 | 30 | 94.0 |
| Rotaclone EIA | Rotavirus | Stool/swab | 45 | 19 | 19 | 26 | 26 | 100 |
| Lactoferrin CHEK | (Qualitative) | Raw | 147 | 41 | 46 | 106 | 101 | 96.6 |

*****Preserved stool specimen types include stools preserved in Cary-Bair (CB), Total-Fix, low viscosity polyvinyl-alcohol (LV), or 10% Formalin, etc. Raw (unpreserved) stools were fresh stool samples within stability time.

**Supplemental TABLE S5** Stool specimen stability in Cary-Blair (C/S) for *Campylobacter* EIAs

1. Refrigerated (2-8 °C) stool stability in Cary-Blair for *Campylobacter* EIAs

| Sample# | Culture | Baseline EIA OD Reading | | | EIA OD Reading post storage | | | | |
| --- | --- | --- | --- | --- | --- | --- | --- | --- | --- |
|  |  | Meridian EIA | Campy Chek EIA | Result | Storage (day) | Meridian EIA | Campy Chek EIA | Result | Stability |
| 1 | + | 3.00 | 3.00 | + | 4 | 3.00 | 3.00 | + | Yes |
| 2 | + | 3.00 | 2.48 | + | 4 | 3.00 | 2.77 | + | Yes |
| 3 | + | 3.00 | 3.00 | + | 4 | 3.00 | 3.00 | + | Yes |
| 4 | + | 0.36 | 2.10 | + | 4 | 0.69 | 2.45 | + | Yes |
| 5 | + | 3.00 | 3.00 | + | 4 | 3.00 | 3.00 | + | Yes |
| Overall |  | 2.47 | 2.72 |  | 4 | 2.54 | 2.84 |  | Yes |

1. Frozen (-10 ~ -30 °C) stool stability in Cary-Blair for *Campylobacter* EIAs

| # | Culture | Baseline EIA OD Reading | | | EIA OD Reading post storage | | | | |
| --- | --- | --- | --- | --- | --- | --- | --- | --- | --- |
|  |  | Meridian EIA | Campy Chek EIA | Result |  | Storage (day) | Campy Chek EIA | Result | Stability |
| 1 | + | 3.00 | 2.24 | + |  | 14 | 3.00 | + | Yes |
| 2 | + | 2.40 | 2.08 | + |  | 14 | 2.77 | + | Yes |
| 3 | + | 3.00 | 3.00 | + |  | 14 | 3.00 | + | Yes |
| 4 | + | 0.35 | 1.82 | + |  | 14 | 2.45 | + | Yes |
| 5 | + | 3.00 | 3.00 | + |  | 14 | 3.00 | + | Yes |
| Overall | | 2.35 | 2.45 |  |  | 14 | 2.84 |  | Yes |

A


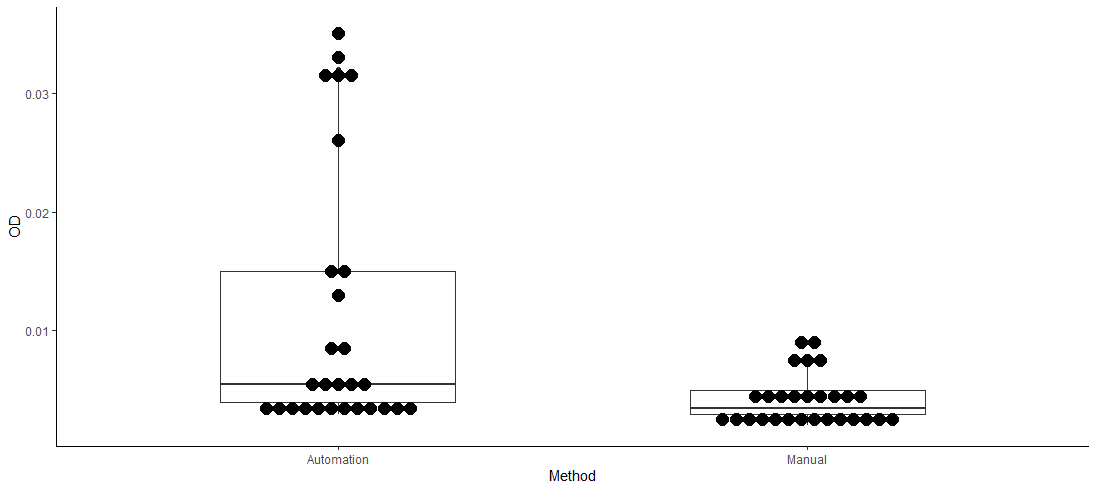


B


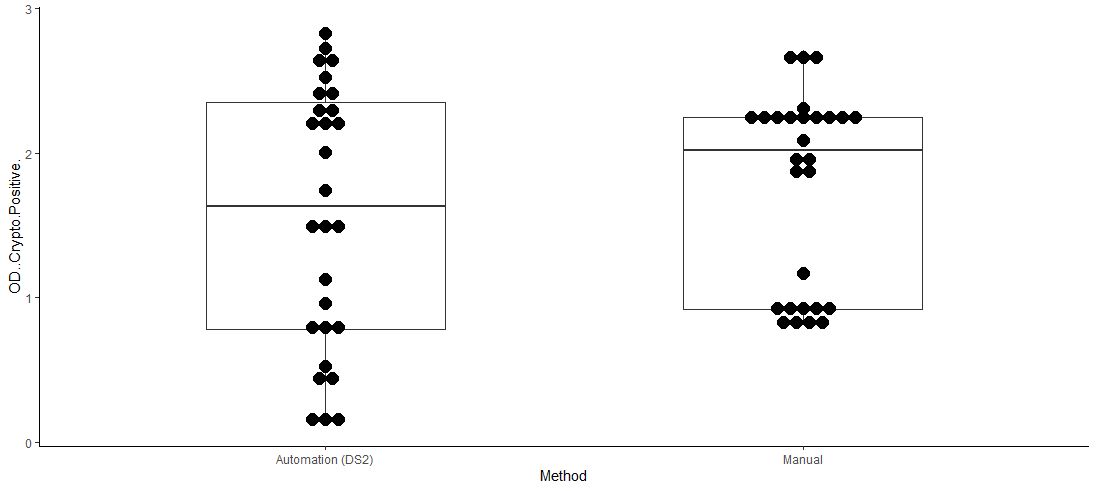


**Supplemental FIG SF1** *Cryptosporidium* EIA OD reading based on manual method and automation method. **A**. Negative samples (n=43). **B**. Positive samples (n=35).
